# Increasing cataract surgical output: my experience at Sabatia Eye Hospital, 2005–2011

**Published:** 2022-12-16

**Authors:** Demissie Tadesse Bekele

**Affiliations:** 1 Regional Eye Health Adviser: CBM Africa East & South Regional Office, Addis Ababa, Ethiopia.

**Improved systems, a supportive environment, and a focus on getting more patients (and treating them more quickly) helped Sabatia Eye Hospital to more than triple its cataract surgical output in a six-year period.**

**Figure:**
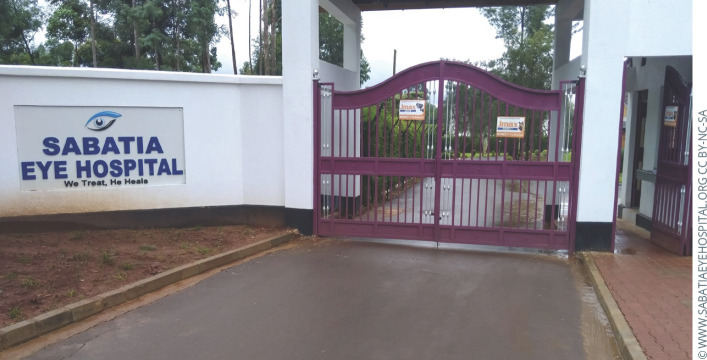
Sabatia Eye Hospital. **KENYA**

Sabatia Eye Hospital, located in a small rural village in Vihiga county, Kenya, is the main eye hospital serving western Kenya, an area with a population of over 4 million people.

When I joined the hospital as medical director in 2005, it was performing an average of 1,500 cataract operations a year – far below its potential, and also far below the number needed to serve the patients in its catchment area (see panel). Sabatia’s financial situation was also precarious.

To enable the hospital to offer high-quality, high-volume eye care services, particularly cataract surgery, we had to work hard to put in place suitable administrative, human resources, financial, and management systems and create a supportive environment where everyone could work together as a team.

**Target cataract surgical output**

How can we determine how many cataract operations an eye unit should aim to perform per year? This is known as the ‘target cataract surgical rate’ and is calculated by multiplying the clinic’s catchment population by the cataract surgical rate per million population needed to reduce visual impairment due to cataract in that region or country.

A study carried out in 2013 estimated that the target cataract surgical rate needed to eliminate cataract visual impairment in Africa (at the level of 6/18) ranges between 1,200–4,500 operations per year per million population.^1^ This figure is based on estimates of the prevalence of visual impairment due to cataract in a population (e.g., using a Rapid Assessment of Avoidable Blindness or RAAB survey), as well as the number of new cases of cataract that is expected to develop in each population every year (the incidence). Incidence depends on many factors, including the age structure of the population.

To estimate the cataract surgical rate needed in Western Kenya, therefore, multiply the catchment population (approximately 4 million) by the estimated cataract surgical rate per million population (1,200-4,500). This gives an estimated target cataract surgical rate of between 4,800 and 18,000 operations per year for the region.

Even with full support from hospital staff members, the hospital board, and the international non-governmental organisation CBM, it took almost a year to develop and strengthen these systems and get the hospital on the right track.

Our first aim was to increase the number of cataract operations per year, while maintaining quality. To achieve this, we needed to have more patients and treat them more quickly.

We made three key immediate changes to our policies and ways of working:

1. The outpatient clinic was kept open during working hours (instead of just a few hours per day).
2. Cataract patients arriving at the clinic without appointments (i.e., as ‘walk-ins’), whether referred by someone or on their own, were admitted and even operated on that same day, provided they were fit and willing to undergo surgery.
3. Patients were discharged the next day, which went against the trend of keeping them in the hospital for several days.

However, we knew that we needed to do more.

## Outreach services

As Sabatia is in a rural area, it is not easily accessible. To improve access, particularly for people in under-served areas and communities, the hospital developed partnerships with churches, hospitals, health centres, companies, and community organisations in the surrounding areas. These helped us by:

- Talking to the community about cataract and encouraging them to come for surgery (‘mobilisation’) using different means, such as via local radio programmes, at church gatherings, and at public events and spaces, such as market places.
- Sponsoring operations. Local companies and Lions clubs provided funding to cover the costs of patients who could not afford surgery.
- Volunteering at outreach events by helping us with screening, referring, and making appointments for patients who need surgery
- Offering spaces to serve as outreach surgical facilities when we held surgical camps in health centres, district hospitals, and in schools.
- Offering their employees’ time to support the hospital team in the following areas: counselling patients and making appointments for them, admissions, the operating theatre, and postoperative follow-up.

Intensifying efforts and strengthening the output and quality of the base hospital and outreach services helped to build a **better reputation** for the hospital. This allowed us to become increasingly well known as a major eye care service provider in western Kenya. This not only increased the number of patients coming for eye care services, but also helped to develop the capacity of the hospital by bringing in more material and financial support from charities and others. Whereas the cost of surgery was initially subsidised by charities, the revenue generated thanks to the increased number of patients could be used to further improve quality, buy equipment, and subsidise patients who could not afford surgery.

## Primary eye care training

The hospital started its own primary eye care training programme (an ophthalmic skills upgrading course) in order to establish primary eye care services at health centres and district hospitals in the catchment area. This has helped in **identifying and referring** more patients with cataract to the base hospital and outreach locations. It also improved patient mobilisation for surgery, facilitated better follow-up, and significantly improved cataract surgical output and outcomes.

All these strategies and efforts improved our annual surgical output to over 5,000 operations per year in 2011. At the same time, the centre generated more revenue, which further helped us to motivate staff members, buy better quality equipment, develop better infrastructure, carry out more cataract operations with better outcomes, and expand the eye care service.
